# Thioredoxin Downregulation Enhances Sorafenib Effects in Hepatocarcinoma Cells

**DOI:** 10.3390/antiox8100501

**Published:** 2019-10-22

**Authors:** María José López-Grueso, Raúl González, Jordi Muntané, José Antonio Bárcena, C. Alicia Padilla

**Affiliations:** 1 Department of Biochemistry and Molecular Biology, University of Córdoba, 14071 Córdoba, Spain; q02logrm@uco.es (M.J.L.-G.); bb1papec@uco.es (C.A.P.); 2 Institute of Biomedicine of Seville (IBiS), Hospital University “Virgen del Rocío”/CSIC/University of Seville, 41013 Sevilla, Spain; b62goojr@uco.es (R.G.); jmuntane-ibis@us.es (J.M.); 3 Departamento de Cirugía General, Hospital Universitario Virgen del Rocío/Instituto de Biomedicina de Sevilla (IBiS)/CSIC/Universidad de Sevilla, 41013 Sevilla, Spain; 4 Centro de Investigación Biomédica en Red de Enfermedades Hepáticas y Digestivas (CIBERehd), 41013 Sevilla, Spain; 5 Instituto Maimónides de Investigación Biomédica de Córdoba (IMIBIC), 14004 Córdoba, Spain

**Keywords:** hepatocarcinoma, thioredoxin, sorafenib, redox signaling, EMT

## Abstract

Sorafenib is the first-line recommended therapy for patients with advanced hepatocarcinoma (HCC) in de-differentiation stage (presenting epithelial–mesenchymal transition, EMT). We studied the role of the thioredoxin system (Trx1/TrxR1) in the sensitivity or resistance of HCC cells to the treatment with Sorafenib. As a model, we used a set of three established HCC cell lines with different degrees of de-differentiation as occurs in metastasis. By quantitative proteomics, we found that the expression levels of Trx1 and TrxR1 followed the same trend as canonical EMT markers in these cell lines. Treatment with Sorafenib induced thiol redox reductive changes in critical elements of oncogenic pathways in all three cell lines but induced drastic proteome reprograming only in HCC cell lines of intermediate stage. Trx1 downregulation counteracted the thiol reductive effect of Sorafenib on Signal Transducer and Activator of Transcription 3 (STAT3) but not on Mitogen-Activated Protein Kinase (MAPK) or Protein Kinase B (Akt) and transformed advanced HCC cells into Sorafenib-sensitive cells. Ten targets of the combined Sorafenib–siRNATrx1 treatment were identified that showed a gradually changing expression trend in parallel to changes in the expression of canonical EMT markers, likely as a result of the activation of Hippo signaling. These findings support the idea that a combination of Sorafenib with thioredoxin inhibitors should be taken into account in the design of therapies against advanced HCC.

## 1. Introduction

Hepatocarcinoma (HCC) represents 80% of the primary hepatic neoplasms that appear mainly in a context of chronic liver cirrhosis. It is the sixth most frequent neoplasm, the third cause of cancer death, and accounts for 7% of registered malignancies [1]. Sorafenib is the standard of care for advanced-stage HCC, as demonstrated in two large-scale trials [2] and the Asia-Pacific trial [3].

Epithelial–mesenchymal transition (EMT) is an important process that happens in normal development in which epithelial cells lose many of their properties to become mesenchymal cells by means of drastic changes in architecture and behavior. A similar transition also occurs during tumor progression and malignant transformation, leading to increased cell motility and invasiveness. Transforming Growth Factor-ß (TGFB) is known as the main, although not exclusive, inductor of EMT, which takes place through various signaling pathways [4]. A critical molecular event of EMT is the downregulation of the cell adhesion molecule E-cadherin and its replacement by N-cadherin [5]. Activation of Akt leads to a significant reduction in E-cadherin expression and to nuclear localization of SNAI1, suggesting a role for the PI3K/Akt signaling pathway in the shift from E-cadherin to N-cadherin expression and in EMT progression in cancer [6,7]. The serine/threonine kinase Akt is thought to be responsible for mediating the acquired resistance to Sorafenib in HCC cells [8]. Akt can be activated by insulin and survival and growth factors through phosphorylation mediated by mTOR2 and PDK1 at specific threonine residues. The process can be reverted by protein phosphatase 2 (PP2A) when Akt is oxidized, suggesting that Akt is subjected to redox regulation [9]. Moreover, it was shown that the interaction between Akt and PP2A under conditions of oxidative stress could be impaired by the reduction of a specific disulfide bond in Akt catalyzed by glutaredoxin (Grx). Activation of Akt after stimulation with insulin and various growth and survival factors, leading to the phosphorylation at Thr308 and Ser473 by PDK1 and mTOR2, respectively. Akt in its oxidized form can also be inactivated by protein phosphatase 2 (PP2A) dephosphorylation, which suggests that Akt is a redox-regulated protein [9]. It has been shown that Grx prevented Akt from forming a specific disulfide bond between Cys-297 and Cys-311 and suppressed its association with PP2A under oxidative stress, [9,10,11].

STAT3 is a pro-survival transcription factor which is constitutively activated in human cancer cell lines. Constitutive phosphorylation of STAT3 causes important changes in apoptosis, angiogenesis, invasion, migration, and proliferation, resulting in cell malignant transformation [12]. It has been observed that STAT3 activation is involved in EMT, invasion, and generation of metastasis in HCC [13]. Tyrosine phosphorylation of STAT3 is dependent on the thiol redox state modulated by H_2_O_2_ and Prx2 levels and the thioredoxin system activity [14]. Trx1–STAT3 disulfide exchange intermediates were detected, suggesting that Trx1 may be a direct mediator of STAT3 disulfide reduction.

Overexpression of Trx1 has been detected in human cervical neoplastic squamous epithelial cells, lung, colon, and HCC tumors [15,16,17,18]. Targeting Trx1 for cancer therapy and as prognostic marker of HCC has been considered because of the observed relationship between Trx1 expression and tumor aggressiveness, although the mechanisms underlying this association are still not well known [19]. Overexpression of Trx1 is one of the mechanisms for drug resistance in HCC treatment. A possible strategy that is gathering strength is to combine Trx1 gene therapy with chemotherapy to increase the effectiveness of treatments in HCC [20]. Thioredoxin-interacting protein (TXNIP) is a member of the alpha-arrestin protein family that binds to the active site and counteracts the action of Trx1 functioning as tumor suppressor [21]. Downregulation of TXNIP exacerbates cancer progression, and its expression is actually reduced in various human cancer cells [22]. TXNIP deficiency enhances the induction of the Zinc finger proteins SNAI1 and SNAI2 by TGF-ß and promotes TGF-ß-induced EMT [23].

A comparative genomic characterization of 19 cell lines derived from HCC allowed clustering them into two groups (I and II) according to their gene expression patterns [24].

In this study, we selected three hepatocarcinoma cell lines, i.e., HepG2 belonging to group I and SNU423 and SNU475 belonging to group II, to carry out a comparative study of their basal proteome and of how it responds to Sorafenib treatment and/or thioredoxin downregulation. We also analyzed redox modifications and phosphorylation of Akt, MAPK, and STAT3. The results obtained support the use of a combination of Sorafenib with Trx1 inactivation in HCC therapy.

## 2. Materials and Methods

### 2.1. Materials

HepG2 (HB-8065™, ATCC/LGC Standards, SLU, Barcelona, Spain), SNU423 (CRL-2238, ATCC/LGC Standards) and SNU475 (CRL-2236, ATCC/LGC Standards) cell lines were obtained from American Type Culture Collection (ATCC; LGC Standards, S.L.U., Barcelona, Spain). Anti-Trx1 was obtained in-house from rabbit immunization with Trx1. Antibodies against STAT3 (#4904S), MAPK (#9102), Thr^202^/Tyr^204^pMAPK (#4370P), Ser^473^pAkt (#4060S) were obtained from Cell Signaling Technology. Antibodies against Tyr^705^pSTAT3 (#sc-7993), TXNIP (#sc-271237), Akt1 (#sc-5298), and ß-actin (#sc-47778) were from Santa Cruz Biotechnology, Inc. (Dallas, TX, USA). Anti-TrxR1 (#ab124954) was provided by Abcam, Inc. Secondary antibodies were from Sigma. ECL reagent was from GE Healthcare (Chicago, Illinois, USA); siRNA for Trx1 was from Dharmacon, Inc. (#L-006340-00; Lafayette, Colorado, USA).

### 2.2. Cell Growth Conditions

Cell cultures were negative for mycoplasma contamination. Cells were maintained in Eagle’s minimum essential medium (EMEM), pH 7.4, supplemented with 10% FBS (#S181G-500, Biowest), sodium pyruvate (1mM) (#L0642-100, Biowest), and a penicillin–streptomycin–amphotericin solution (100U/mL–100μg/mL–0.25 µg/mL) (#L0010-100, Biowest) at 37 °C in a humidified incubator with 5% CO_2_. The cells were seeded at a density of 10^5^ cells/cm^2^ in 2D culture. When applied, Sorafenib was added to the cell culture at a concentration of 10 µM, as set up in a previous study [25] and the cells were incubated for 24 h before cell lysis to measure cell proliferation and protein activities and expression and to perform proteomic analysis. Cells lysis was carried out using 50 mM HEPES pH 7.5, 5 mM EDTA, 150 mM NaCl, 1% NP-40, a proteases inhibitor cocktail (#P8340, Sigma-Aldrich), 1 mM phenylmethylsulfonyl fluoride (PMSF), 1 mM NaF, and 1mM Na_3_VO_4_. The lysates were incubated on ice and vortexed for 15 s in four intervals of 5 min each. The samples were centrifuged at 13,000 rpm at 4 °C. The supernatants were collected for protein quantification and analysis.

### 2.3. Determination of Proliferation and Caspase-3 Activity Measurement

Cell proliferation was analyzed using a colorimetric ELISA (Roche Applied Science, Penzberg, Germany). In total, 2 × 10^4^ cells/cm^2^ were cultured in 96-well plates at 37 °C and 5% CO_2_. At the end of the experiment, the cells were incubated with a 10 µM BrdU labelling solution for 6h at 37 °C following the protocol recommended by the manufacturer. Caspase-3-associated activity was determined using the peptide-based substrate Ac-DEVD-AFC (100 µM), as described elsewere [10].

### 2.4. SDS-PAGE and Western Blotting

The protein expression levels of Trx1, TrxR1, TXNIP, STAT3, pSTAT3, Akt1, pAkt, MAPK, and pMAPK were determined by SDS-PAGE coupled to Western blotting analysis, following the same procedure and using the same antibody dilutions as described before [10].

### 2.5. Silencing of Trx1

Human Trx1 was knocked down in HepG2, SNU423, and SNU475 cells using a pool of four specific siRNA in 6-well plates (20,000 cells/cm^2^), according to the manufacturer’s recommendations (Dharmacon, GE Healthcare Life Sciences) and as described before for HepG2 cells [10]. Non-targeting negative controls were run. Silencing of Trx1 was always checked by Western blotting and activity assay to confirm that its levels were reduced by ≈80%.

### 2.6. Redox Mobility Shift Assay

Detection of thiol redox changes in Akt, MAPK, and STAT3 proteins was performed by differentially labeling reduced cysteines with 5 mM N-ethylmaleimide (NEM) and reversibly oxidized cysteines by reduction with 5 mM tris (2-carboxyethyl) phosphine (TCEP), followed by labeling with 10 mM 4-acetamido-4’-maleimidylstilbene-2,2’-disulfonic acid (AMS) (Thermo Scientific Pierce), which increases the molecular weight of the native protein by 536.44 Da. The protocol was the same as described before [10].

### 2.7. LC–MS/MS, Label-Free MS Protein Quantification, and Systems Analysis

Proteomic analyses were performed at the Proteomics Facility, University of Córdoba (SCAI). The procedure started with a culture of 2 × 10^4^ cells/cm^2^, and the protocol was the same as described previously [26]. Peptides were scanned and fragmented with the Thermo Orbitrap Fusion (Q-OT-qIT, Thermo Scientific) equipped with a nano-UHLC Ultimate 3000 (Dionex-Thermo Scientifics). The analysis of MS raw data was performed using the MaxQuant (v1.5.7.0) software [27] and the label-free quantification configuration described before [26]. Three RAW data files per sample from three separate experiments were analyzed. Human UniProtKB/Swiss-Prot protein database (February 2018 version) was searched for protein identification as described before [26]. The method used for the imputation of missing values was the mean imputation. Finally, only the conditions with 3 values per identification were considered and analyzed through ANOVA and Tukey’s post-hoc tests. A protein was considered differentially expressed if identified and quantified with at least 2 unique peptides and had a fold change of at least 1.50 and *p* value ≤0.05.

The differentially expressed proteins together with the fold change values were analyzed with the online IPA software package (Qiagen, version 48207413) and the open software DAVID [28]. For IPA, each protein was mapped to its corresponding gene object in the Ingenuity Pathways Knowledge. The “core analysis” function was carried out considering direct and indirect relationships experimentally observed in all mammalian tissues and species, as well as all node types, data sources, and mutations. The list of significantly enriched canonical pathways, biological functions, and upstream factors is presented together with the activation or inhibition z-score values in a scale of colors.

### 2.8. Statistical Analysis

Results are expressed as mean ± SD of data from ≥3 independent experiments. One-way ANOVA with the least significant difference Tukey’s test as post-hoc multiple comparison analysis with a single pooled variance was used for comparisons; output *p* value ranges were handled in GP style: 0.0332 (*), 0.0021 (**), 0.0002 (***), <0.0001 (****). The threshold for statistically significant differences was set at *p* adjust ≤0.05 value.

## 3. Results

### 3.1. Tracing the Proteomic Signature of EMT in Human Hepatocarcinoma Cell Lines

**Figure 1:**
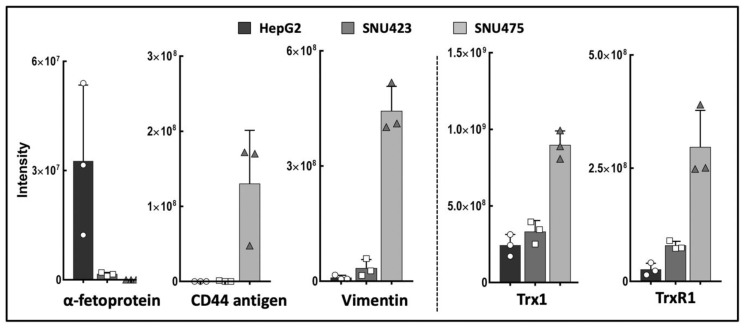
Epithelial–mesenchymal transition (EMT) markers and thioredoxin system in three hepatocarcinoma (HCC) cells lines. Data for vimentin, alpha-fetoprotein, CD44 antigen, Trx1, and TrxR1 were retrieved from the quantitative proteomic analysis of HepG2, SNU423, and SNU475 cells (Supplementary File S1). The scale in the vertical axis is the relative intensity from the LC–MS/MS quantitative analysis and varies between proteins; the maximum value for each protein ranges from 3.25e + 007 for alpha-fetoprotein in HepG2 cells to 8.97e + 008 for Trx1 in SNU475 cells. (*N* = 3, individual values are shown).

A “label-free” quantitative proteomics analysis detected 1170 proteins with significant differences between HepG2, SNU423, and SNU475 cell lines (Supplementary File S1). This is the first comparative proteomic analysis carried out with these cell lines, and the results obtained for canonical markers of EMT showed increasing and decreasing gradients, in agreement with the classification of these human HCC cells as epithelial or mesenchymal [29,30]. These results strongly correlate with previous microarray and Western blotting analyses of human HCC cell types [29,30] and constitute a definitive proof of concept for our experimental approach. E-cadherin was not detected, likely because our proteomic protocol was not optimized for membrane proteins. The members of the Trx system Trx1 and TrxR1 were also present in increasing levels from HepG2 to SNU475 cells (Figure 1), which agrees with the role described for Trx1 as a pro-metastasis factor [31].

**Figure 2:**
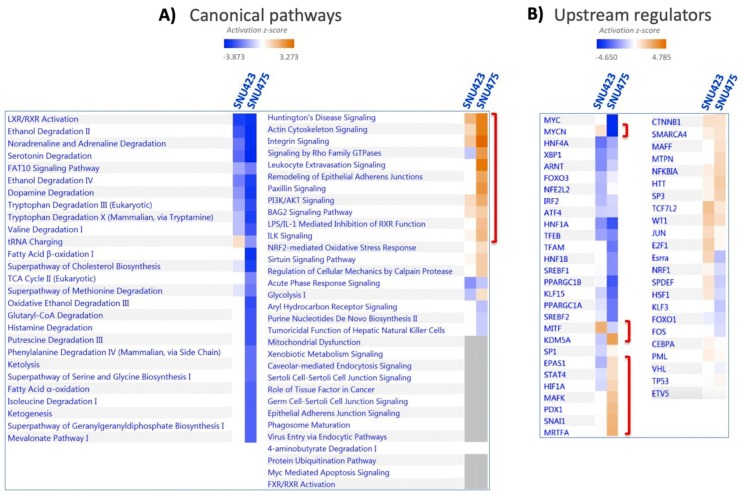
Systems Biology analysis of differential proteomic data from three HCC cell lines. Quantitative data on protein levels in SNU423 and SNU475 cells relative to the levels in HepG2, the more differentiated cell line, were analyzed. Up- and down-regulated proteins were analyzed using the IPA software in terms of (**A**) canonical pathways based on the content of the Ingenuity Knowledge Base, and (**B**) Upstream Regulator Analysis to identify the cascade of upstream transcriptional regulators (see M&M Section 2.7 for more details). The degree of activation is represented in a color scale as indicated. Red brackets have been added to highlight significant data commented on in the main text.

A system analysis of these 1170 differential proteins yielded significant enrichments in several canonical pathways (Figure 2A; the identities of the proteins are shown in Supplementary File S1). Integrin and actin cytoskeleton signaling, remodeling of epithelial adherent junction, and PI3K/Akt signaling, which have been described as being involved in EMT, were activated. On the reverse side, there was an overall inactivation of amino acid metabolism, fatty acid beta-oxidation, neurotransmitter catabolism, glutathione metabolism, and oxido-reduction processes. SNU423 and SNU275 cells showed similar activation and inactivation trends in many processes, although these processes were affected to a lesser extent in SNU423 cells, in parallel to their degree of mesenchymal properties. System analysis of 100 upregulated and 156 downregulated proteins common to the first two cell lines by Kyoto Encyclopedia of Genes and Genomes (KEGG) pathways and Gene Ontology (GO) biological processes using DAVID software confirmed and stressed these similarities (Figure S1). A search for upstream regulators brought to light differences and commonalities (Figure 2B). To name a few, MYCN and MITF transcription factors appeared to be activated in SNU423 but inactivated in SNU475 cells, whereas HIF1A and the KDM5A histone H3 demethylase behaved in the opposite way.

Altogether, these results identified a number of proteins and regulatory elements related to de-differentiation of HCC cells that could be an interesting focus for further research.

### 3.2. The Trx/TrxR/TXNIP System is Highly Sensitive to Sorafenib Treatment in HCC Cell Lines

**Figure 3:**
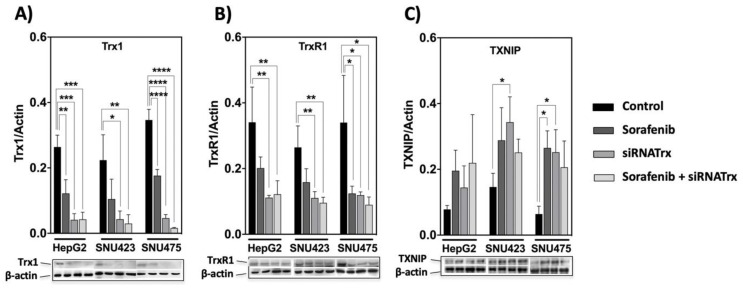
Response of the thioredoxin system to Sorafenib and siRNATrx1 treatments. Changes in the levels of three components of the thioredoxin system, (**A**) Trx1, (**B**) TrxR1 and (**C**) TXNIP were determined in HepG2, SNU423 and SNU475 cells by Western blotting with their specific antibodies on treatment with Sorafenib or siRNATrx1 independently or combined under the conditions described in M&M. Densitometric data normalized for ß-actin are shown; samples from 3 different experiments (*N* = 3) for each cell line were run in the same gel; protein levels between cells cannot be compared since each set of 3 replicas for each cell line was developed on a different WB membrane; for a comparison of Trx1, TrxR1 between cell lines, refer to quantitative proteomic data in Figure 1; a composition of representative blots is shown below each graph. *p* values 0.0332 (*), 0.0021 (**), 0.0002 (***), <0.0001 (****), see Materials & Methods for statistical details.

The basal levels of Trx1 and TrxR1 in control cells were markedly higher in mesenchymal SNU475 cells (Figure 1), but Sorafenib induced a significant decrease in their levels in all HCC cell lines (Figure 3A,B). TXNIP behaved in a reciprocal manner relative to Trx1 and TrxR1. It was upregulated both by Sorafenib and by siRNATrx1, either independently or jointly, in all three cell lines (Figure 3C). These results indicate that the downregulation of the Trx system is part of the antitumoral action mechanism of Sorafenib and support our hypothesis that co-treatment with Trx1 inactivation adjuvants would potentiate Sorafenib-based antitumoral therapy.

### 3.3. The Effect of Sorafenib on the Global Proteome Depends on the Cell De-Differentiation Stage

**Figure 4:**
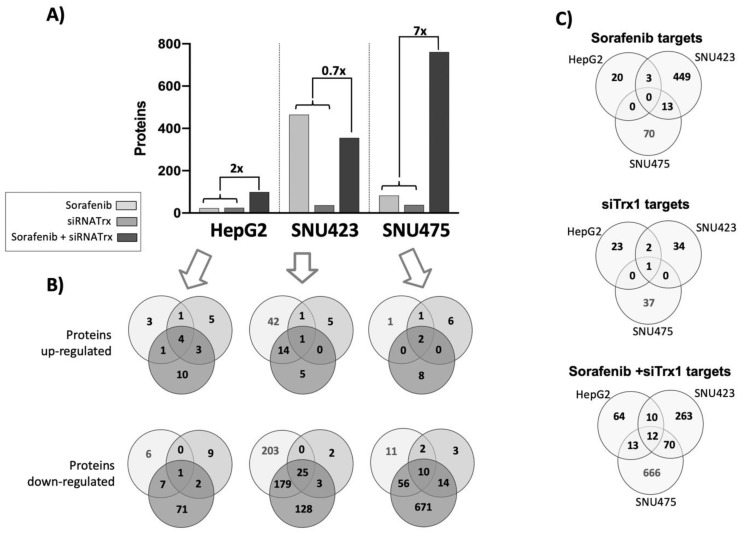
Summary of the quantitative proteomic analysis of HCC cell lines subjected to Sorafenib and/or siRNATrx1 treatments. (**A**) The total number of significantly (adjusted *p* ≤ 0.05) different proteins (fold change ≥1.5 and ≤0.6) quantified upon each treatment relative to their controls; the numbers above the brackets and thin lines are the “synergy factor” reached by siRNATrx1 and Sorafenib calculated by the ratio between the number of proteins affected by the combined treatment and the number of proteins affected by individual Sorafenib and siRNATrx1 treatments. (**B)** Venn diagrams of upregulated and downregulated proteins by each treatment, by cell line; the color code indicated in the legend applies to (A) and (B). (**C**) Venn diagrams of proteins affected in each cell line by treatment.

Sorafenib alone was very effective in SNU423 cells, inducing significant changes in 465 proteins, whereas in HepG2 and SNU475 cells, the number of proteins affected was restricted to only 23 and 83, respectively (Figure 4A). The effect of siRNATrx1 alone was rather low in all three cell types, as should be expected since the effects of this antioxidant protein at the transcriptional regulatory level are limited. The cellular environment determined which proteins were affected by Trx1 downregulation, since there were no common siRNATrx1 targets among the three cell lines, although most of the proteins affected did also change when siRNATrx1 treatment was combined with Sorafenib (Supplementary File S1). Interestingly, a very strong synergistic effect was observed when Sorafenib was applied to Trx1-downregulated SNU475 cells (Figure 4A). This synergistic effect was also observed in HepG2 cells, although to a lesser extent, but was absent in SNU423 cells.

Altogether, these proteomic data indicate that Sorafenib is highly active in moderately differentiated tumor cells but it has a limited effect in Trx1-rich and poorly differentiated cells, unless the antioxidant thioredoxin-related signaling is weakened. Its action is minor in epithelial HepG2 cells even in combination with Trx1 downregulation.

**Figure 5:**
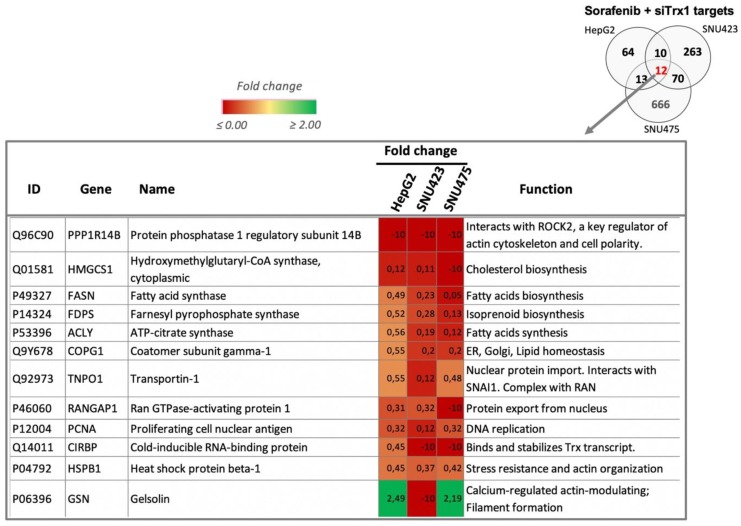
Common target proteins of the Sorafenib + siRNATrx1 treatment. Twelve proteins that changed significantly in HepG2, SNU423, and SNU475 cells are shown. A brief description of their function was extracted from UniProt; fold changes relative to their respective controls treated with solvent + Non-target siRNA are indicated in a color scale. Quantitative proteomic analysis as described in Materials and Methods (*N* = 3). Tenfold-change values correspond to proteins that were present in the controls but were not detected in the treated samples.

A comparison of the Sorafenib and Trx1 silencing targets of each cell line (Figure 4C) highlighted the fact that there were no common targets between the se cell lines, suggesting that the effects of each treatment are dependent on the de-differentiation stage of HCC cell lines. However, there were 12 common targets of the combined treatment in the three cell lines (Figure 4C and Figure 5). These common proteins are related to lipids biosynthesis, protein nuclear transport, and actin organization. The increasing degree of downregulation of most of these common proteins runs parallel to their degree of de-differentiation.

**Figure 6:**
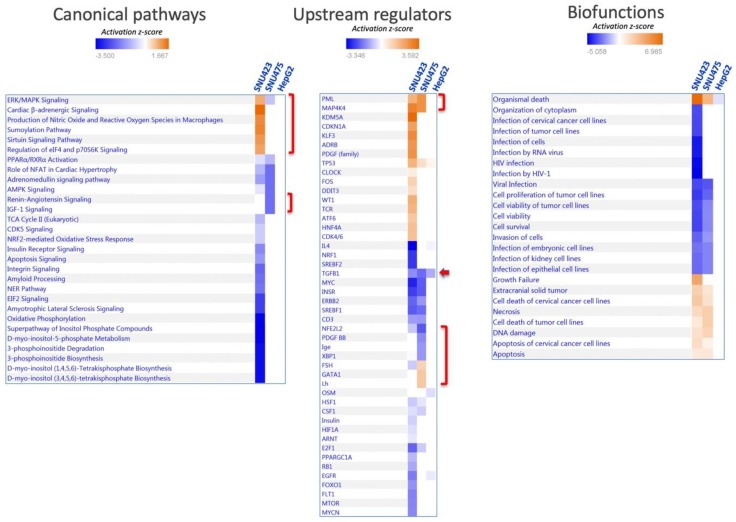
Enrichment analysis of proteins affected by Sorafenib treatment in each cell line. Proteins affected by Sorafenib treatment alone were analyzed using the IPA software in terms of canonical pathways, upstream regulators, and biofunctions as indicated, in the three cell lines studied. Brackets and arrow highlight elements that are commented on in the main text.

System analysis of the proteomic data provided some clues about the differential effect of Sorafenib in each cell type (Figure 6). The only pathways altered by Sorafenib in SNU475 cells were those of IGF-1 and renin–angiotensin signaling, which appeared inactivated with the concomitant differential inactivation of several upstream regulators, i.e., NFE2L2, PDGF-B, a potent mitogen for cells of mesenchymal origin, XBP1, involved in the unfolded protein response (UPR), and Ige (Figure 6). None of these pathways and upstream regulators were affected in SNU423 cells.

Pathways affected by Sorafenib solely in SNU423 cells were those related to integrin and EIF2 signaling, oxidative phosphorylation, and phosphoinositol metabolism, which were inactivated, and to sumoylation and sirtuin, which were activated (Figure 6). In parallel with these changes, a set of upstream regulators were uniquely either inactivated—IL4, SREBF2, NRF1, FOXO1, EGFR, MTOR, MYCN—or activated—KDM5A histone H3 demethylase, KLF3, and PDGF, to name the most relevant.

Despite the striking differential outcomes of Sorafenib treatment between SNU423 and SNU475 cells in terms of pathways and number of proteins affected, several upstream regulators responded equally to Sorafenib in both cell types. Briefly, MAP4K4 and the protein PML, which is regulated by sumoylation, were activated, whereas MYC, E2F1, the growth enhancer and cysteine-rich receptor tyrosine-protein kinase ERBB2, which interacts with STAT3, the steroid regulatory element-binding factor 1 SREBF1, and the insulin receptor tyrosine kinase INSR were inactivated. TGFB1, the main inducer of EMT [4], was the only regulator affected in all the cells and was inactivated even in HepG2.

### 3.4. The Alteration of the Global Proteome by Sorafenib in Human HCC Cell Lines at Different De-Differentiation Stages is Conditioned by Trx1 Downregulation

The number of new proteins affected by Sorafenib under conditions of Trx1 downregulation was 81, 133, and 679 in HepG2, SNU423, and SNU475 cells, respectively (Figure 4B). GO terms enrichment analysis of these newly affected sets of proteins with DAVID software revealed some common biological processes affected in the three cell lines: cholesterol and isoprenoid metabolism, cell–cell adhesion, and proteostasis.

**Figure 7:**
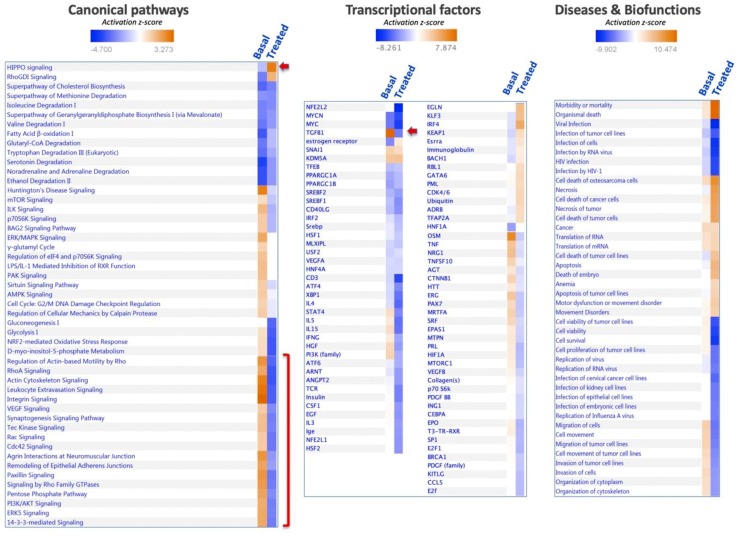
Enrichment analysis of proteins affected by combined treatment of Sorafenib and siRNATrx1 in SNU475 cells. In total, 761 proteins differentially quantified in SNU475 cells upon the combined treatment of Sorafenib and siRNATrx1 were analyzed using the IPA software by canonical pathways, transcriptional factors, and biofunctions. The enrichment results are indicated in colored scale in the right column (“Treated”) in each panel; the left column, headed “Basal”, shows the proteomic signature of SNU475 cells obtained by reference to HepG2 cells in the absence of treatment.

Analysis of the set of proteins affected in SNU475 cells under the combined treatment revealed the target pathways (Figure 7). The general trend was inactivation of most pathways (actin, integrin, 14-3-3-mediated signaling, PI3K/Akt, sirtuin, mTOR, ILK, EIF2 signaling pathways, glycolysis, gluconeogenesis, and pentose phosphate pathway), except for two pathways that were activated: HIPPO and RhoGDI signaling (Figure 7). Despite the profound effect of the Sorafenib+siRNATrx1 treatment on the proteome and pathways of SNU475 cells, only a small number of transcriptional regulators was markedly affected, with TGFB1 inactivation standing out. These changes were paralleled at the level of diseases and biofunctions by the inactivation of cytoskeleton organization, tumoral cell movement, migration, invasion, viability, and survival, and by the activation of cell death (Figure 7)

Activation of the HIPPO signaling pathway by the combined treatment of Sorafenib and siRNATrx1 would restrict cell proliferation and promote differentiation to cell death [32].

### 3.5. Effect of Sorafenib and Trx1-Silencing on Proliferation and Apoptosis in Three HCC Cell Lines

**Figure 8:**
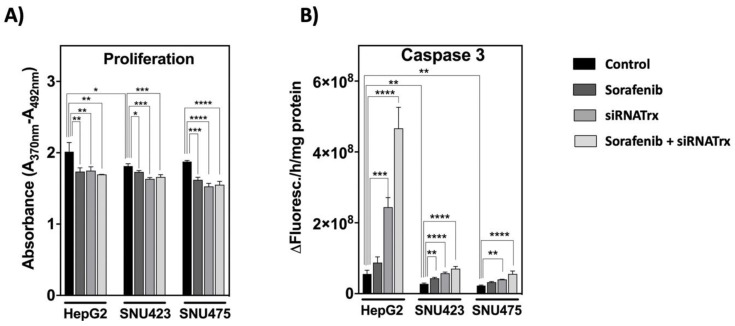
**Proliferation and apoptosis of HCC cell lines subjected to Sorafenib and/or siRNATrx1 treatments.** (**A**) Proliferation as determined by BrdU incorporation into DNA and (**B**) caspase-3 activity determined by fluorescence using a specific peptide substrate. Three different experiments (*N* = 3) for each cell line. *p* values 0.0332 (*), 0.0021 (**), 0.0002 (***), <0.0001 (****), see Materials & Methods for statistical details.

Proliferation under basal conditions was higher in HepG2 cells. Sorafenib and siRNATrx1, either individually or combined, had a significant, non-additive, negative effect on cell proliferation, as determined by BrdU incorporation (Figure 8A). The effect of Sorafenib was lower in SNU423 cells, despite the thorough change induced on the proteome (Figure 4).

Regarding apoptosis (Figure 8B), Trx1 downregulation induced a significant increase in caspase-3 activity in all three cell types, which was inversely proportional to the basal levels of Trx1: prominent in HepG2 cells, as described previously [10], but subtle in SNU423 and SNU475. The effect of Sorafenib alone was slightly significant in SNU423 cells but was markedly enhanced by Trx1 silencing. Again, the enhancing effect was especially prominent in HepG2 cells (Figure 8B).

It seems that the more epithelial HepG2 cells have a programed cell death response to Sorafenib under Trx1 down-regulation aimed at tissue survival, but this response mechanism seems to be impaired in the more mesenchymal cells. The proteomic data showed a decrease in proteins involved in cell proliferation, survival, invasiveness and migration but an increase in proteins involved in cell death in SNU423 and SNU475 cells (Figure 6). Regulated cell death can proceed by mechanisms different from apoptosis and it has been described that Sorafenib induces cell death by ferroptosis in hepatocarcinoma cell lines [33].

### 3.6. Sorafenib Reductive Effect on the Thiol Redox State of Akt, MAPK, and STAT3 Is General but the Counteracting Action of Trx1 Knock-Down is Specific for STAT3

**Figure 9:**
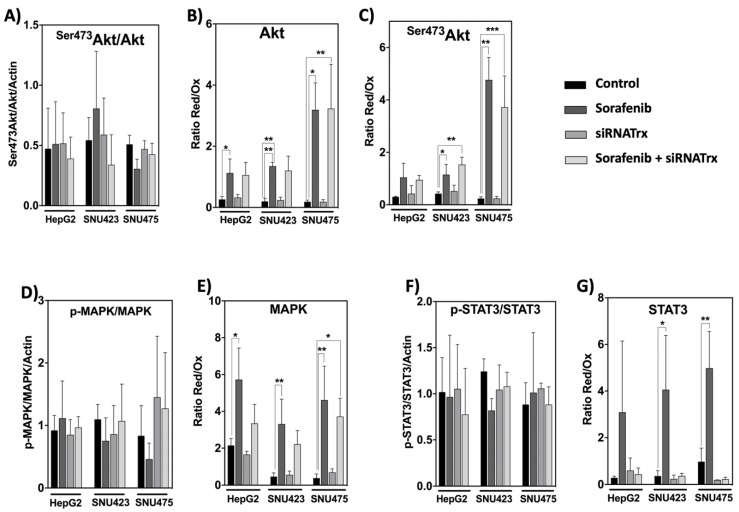
Phosphorylation and thiol redox state of key signaling elements in HCC cell lines treated with Sorafenib and/or siRNATrx1. The phosphorylated/unphosphorylated ratios of the indicated proteins were calculated from the densitometric quantitation data of each form normalized for ß-actin. The ratio Red/Ox reflects the thiol redox state and was calculated from the densitometric data of the bands obtained by the “thiol redox electrophoretic mobility shift assay” as described in Materials and Methods; samples from three different experiments (*N* = 3) for each cell line were run in the same gel; protein levels cannot be compared between cells. *p* values 0.0332 (*), 0.0021 (**), 0.0002 (***), see Materials & Methods for statistical details. (**A**) Ratio of ^Ser473^pAkt/Akt; (**B**,**C**) thiol redox changes of ^Ser473^pAkt and Akt; (**D**,**E**) phosphorylation and redox changes, respectively, of MAPK; the same is shown for STAT3 in (**F**,**G**).

Systems biology analysis of our proteomic data indicated activation of PI3K/Akt signaling in SNU423 and SNU475 cells (Figure 2), but, surprisingly, no significant changes were observed in the ratio between phosphorylated and dephosphorylated Akt, MAPK, and STAT3 in all three cell lines (Figure 9A,D,F). However, Sorafenib treatment provoked a prominent thiol reductive change in Akt, ^Ser473^Akt, MAPK, and STAT3 in all three cell types that was higher for Akt and ^Ser473^Akt in SNU475 cells (Figure 9B,C,E,G). This may be indicative of predominance of redox changes over phosphorylation changes regarding the action of Sorefenib on these signaling pathways. This reductive thiol redox change was insensitive to siRNATrx1, except for STAT3 for which it was totally blunted by Trx1 downregulation (Figure 9G). The involvement of Trx1 in thiol redox changes of STAT3 agrees with the report of the formation of Trx1–STAT3 disulfide exchange intermediates [14], suggesting that Trx1 may be a direct mediator of STAT3 disulfide reduction.

## 4. Discussion

### 4.1. Proteomic Changes during EMT

The current curative treatments of HCC are indicated during the early stages of the disease [34]. Nowadays, however, two-thirds of patients are diagnosed in more advanced stages of the disease [35], urgently requiring new effective and safe drugs. The transition from well-differentiated carcinoma to poorly differentiated invasive carcinoma is a predominant event in the aggressive tumor progression of human HCC. Actually, EMT has been linked to tumor cell invasion and metastasis [36].

We made the first comparative characterization of EMT at the level of expressed proteins in three hepatocarcinoma cell lines, HepG2, SNU423, and SNU475, that present markers indicative of a gradual transition from epithelial to mesenchymal characteristics. A global proteome analysis of these cell lines revealed a thorough change in metabolic processes, consistent with the fact that mesenchymal cells have different metabolic needs compared to epithelial cells, in part because of their motility and invasiveness. Metabolic reprogramming upon EMT in different types of cancer cells is complex and controversial [37], but mitochondrial dysfunction has been widely linked to aggressiveness of cancer and activation of EMT. The finding of a significant decrease in succinate dehydrogenase (SDH) and isocitrate dehydrogenase (IDH) in the Tricarboxylic Acid Cycle (TCA) cycle in the most de-differentiated SNU475 cells (Supplementary File S1), together with a specific activation of a few upstream regulators, including SNAI1 (Figure 2B), agrees with the report that silencing or mutation of both enzymes promoted cell migration and invasion in different types of cancer cells mediated by TGF-ß/SNAI1 [38,39].

Altogether, this first characterization of EMT at a proteome level should help to focus research on events that occur when cancer cells acquire their metastatic and invasive potential and would help in the design of therapies for advanced HCC.

### 4.2. Effect of Trx1 Downregulation on Sorafenib Antitumoral Treatment

Our data show that Sorafenib downregulates specific EMT-related pathways like integrin signaling. However, we found that its proteomic reprogramming capacity is prominent in moderately differentiated SNU423 cells but limited in poorly differentiated SNU475 cells, which represent the most advanced stage of the disease.

Interestingly enough, weakening cell antioxidant defenses by Trx1 downregulation exerted a marked synergistic effect on proteomic rearrangement in Sorafenib-treated SNU475 cells (Figure 4). Sorafenib itself had a down-regulatory effect on the Trx/TrxR1 system and an up-regulatory effect on TXNIP in all three cell types (Figure 3) but only triggered an extensive proteomic reprograming in SNU423, where the basal levels of Trx1/TrxR1 are lower and those of TXNIP might be higher (Figure 1 and Figure 3). However, additional Trx1 downregulation exerted an adjuvant effect that dramatically expanded the action of Sorafenib in SNU475 cells where the basal Trx1/TrxR1 levels are higher and those of TXNIP might be lower. The effect was minor in HepG2 cells.

Although the general significance of these in vitro data in the context of cancer treatment is limited, they strongly support the importance of an anticancer therapy based on a combined treatment of Sorafenib with inhibitors of the Trx system or other prooxidant agents for advanced HCC and encourage the undertaking of further research on this ground.

Few common target proteins were identified in the three cell lines (Figure 4C), likely as a consequence of differences in their cell de-differentiation staging, though several commonalities were obvious at the level of upstream regulators that were affected. A careful characterization of these few common targets may lead to the discovery of true markers for diagnostic and therapeutic purposes. At this point, it is worth mentioning that Protein Phosphatase 1 Regulatory Subunit 14B (PPP1R14B) disappeared from the three cell lines after the Sorafenib + siRNATrx1 combined treatment. PPP1R14B interacts with ROCK2 (“IntAct” EBI-7009696), which is a key regulator of the actin cytoskeleton and cell polarity and is one of the most highly overexpressed phosphatase-related proteins in triple-negative breast cancer (TNBC), which has the worst prognosis among all breast cancers [40].

The only pathway clearly activated by co-treatment was HIPPO signaling, which promotes cell differentiation and contact inhibition of cell proliferation and plays a central role in regulating organ growth and regeneration [32]. It is a critical regulator of liver growth and a potent suppressor of liver tumor formation [41]. Activation of HIPPO signaling by Sorafenib treatment under Trx1 downregulation in poorly differentiated SNU475 HCC cells might impact on the alleviation of EMT and cancer malignancy. To dig deeper into the mechanism, it would be worth checking for possible thiol redox changes in members of the HIPPO signaling pathway that have been reported to be sensitive to the redox environment [42,43].

### 4.3. Involvement of the Trx1-Dependent Thiol Redox State of Key Signaling Pathways

Sorafenib and Trx1 downregulation had a significant negative effect on the proliferation in HCC cell lines independently of their degree of de-differentiation, although their effects were not additive (Figure 8A). Regarding cell death, Trx1 knock-down induced apoptosis in all three cell lines, but the effect was prominent only in differentiated HepG2 cells. The effect of Sorafenib alone was negligible in either cell type, but Sorafenib combined with Trx1 downregulation had a marked pro-apoptotic effect, particularly in HepG2 cells (Figure 8B) and also appreciable in SNU475 cells (Figure 7 and Figure 8B, biofunctions). It seems that the ability of HCC cells to trigger a pro-apoptotic response to Trx1 downregulation and to Sorafenib treatment declines as their basal Trx1 levels and their degree of de-differentiation increase.

The phosphorylation states of Akt, MAPK, and STAT3 were not altered by Sorafenib and/or siRNATrx1 treatments. However, Sorafenib induced a thiol reductive burst that was independent of Trx1 in the case of Akt and MAPK but was totally abolished by Trx1 downregulation in the case of STAT3. These results provide further insight into the importance of redox homeostasis in the mechanism of Sorafenib action and suggest that Sorafenib might protect Akt from PPA2 inactivation by inducing a thiol reductive change, particularly in SNU475 cells. The lack of sensitivity of the thiol redox state of Akt to Trx1 downregulation indicates that this redox change could be mediated by the glutathione system, as had also been previously pointed out in HepG2 cells [10].

Thiol reductive changes in MAPK were also independent of Trx1 silencing but were coincident with the activation of MAP4K4 detected in SNU423 and SNU475 cells treated with Sorafenib alone (Figure 6). The strong enhancing effect of Trx1 silencing on the action of Sorafenib at the proteome level in the poorly differentiated SNU475 cells did not seem to be mediated by phosphorylation or redox changes of Akt and MAPK in the studied cell lines.

Reactive Oxygen Species (ROS) induce tyrosine phosphorylation by inactivation of tyrosine phosphatases and upregulate the DNA binding activity of STAT3 [44]; however, ROS could also induce oxidation of conserved cysteines, impeding STAT3 transcriptional activity [45]. The mechanism of this redox regulation in HepG2 cells involves glutathionylation of STAT3 at Cys328 or Cys542, which are within the DNA-binding domain and the linker domain, respectively, and would affect STAT3 activity under oxidative stress, even when it is phosphorylated at Tyr705 [46,47]. The formation of Trx1–STAT3 disulfide exchange intermediates has been reported, suggesting that Trx1 may be a direct mediator of STAT3 disulfide reduction [14]. The activity of STAT3 must have been affected by the observed redox changes, despite the lack of differences in the degree of phosphorylation, and could have played a role in the response of SNU475 cells to the combined Sorafenib+siRNATrx1 treatment.

Combined treatments of Sorafenib with other agents have been reported. For instance, its combination with metformin downregulated the expression of Trx1 and improved the efficacy of Sorafenib in the treatment of HCC, decreasing tumor invasiveness and cell motility [31], while its combination with tetrandrine was described to induce apoptosis through ROS/Akt signaling in human HCC [48].

## 5. Conclusions

The proteomes of the three HCC cell lines studied reveal differences in proteins involved in motility and invasiveness, coherent with their EMT markers outfit. The present study shows that Sorafenib treatment induces the highest proteomic reprogramming in SNU423 cells whose Trx1/TrxR1 levels are lower and TXNIP levels are higher. However, its effect was limited in poorly-differentiated HCC SNU475 cells, which are expected to be present at high proportion in the advanced stage of the disease and whose basal Trx1/TrxR1 levels are high. However, application of Sorafenib combined with Trx1 knock-down, dramatically enhances proteomic rearrangement, including inactivation of TGFß and activation of HIPPO signaling, accompanied by thiol oxidative changes in STAT3. Combination of Sorafenib with thioredoxin inhibitors should be taken into account in the design of anticancer therapies

## Supplementary Materials

The following are available online at https://www.mdpi.com/2076-3921/8/10/501/s1, Figure S1: System analysis of differential proteins common to SNU423 and SNU475 cells. (A) Proteins upregulated or downregulated in both cell lines relative to HepG2 cells; (B) common proteins from each set were analyzed for enrichment by KEGG pathways and GO biological processes using DAVID software; number of proteins on the right side of the bars refers to the number of proteins clustered in each term; adjusted *p* value is shown on the horizontal axis in a logarithmic scale for statistical significance; Supplementary File S1. Excel file showing the results of quantitative proteomic analysis of HepG2, SNU423, and SNU475 cells. The 1^st^, 2^nd^, and 3^rd^ sheets, (“HepG2 treatments”, “SNU4232 treatments”, and “SNU475 treatments”) show protein ID, number of unique peptides, and protein name in columns A–C. Columns D–O show the normalized abundance for each of three replicates of untreated (“control”), Sorafenib-treated (“Sorab”), siRNATrx1-treated (“Trx1”), and Sorafenib + siRNATrx1 combined treatment (“ST”); columns P–U show the abundance fold change (“FC”) relative to the control and their respective statistical significance (*p*-values, a value of “1” means not significant FC). In the 4^th^ sheet, (“Cell lines”), columns A–D show protein ID, protein name, gene name, and number of unique peptides; columns E–M show the normalized abundance for each of three replicates of untreated HepG2 cells (“HepG2”), SNU423 cells (“S3”), and SNU475 cells (“S5”); columns N–Q show the protein abundance fold change of SNU423 and SNU475 cells (“FCS3_HepG2” and “FCS5_HepG2”) relative to HepG2 together with the statistical significance index (p value). A value of “1” means not significant FC.
