# Supplementary figures and images for: Thioredoxin Downregulation Enhances Sorafenib Effects in Hepatocarcinoma Cells
Source: Antioxidants (Basel). 2019 Oct 22;8(10):501. doi: 10.3390/antiox8100501 (PMC6826379; doi:10.3390/antiox8100501)

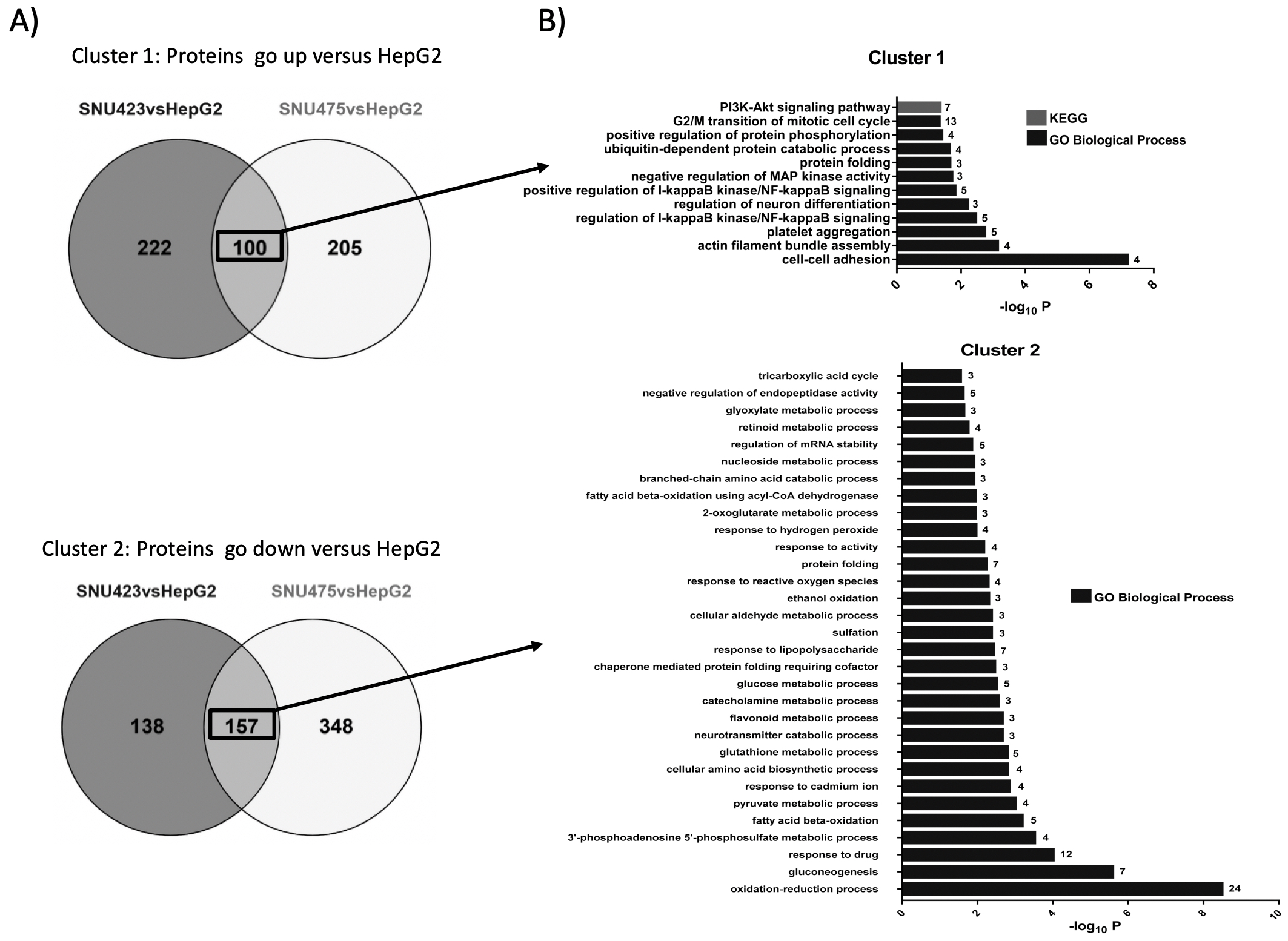

Supplement: Supplementary file 1 [file antioxidants-08-00501-s001.zip › antioxidants-604049-supple/Suppl. Figure 1.jpg]
